# Profile of victims of occupational accidents involving exposure to biological material notified in the city of Palmas, state of Tocantins, Brazil

**DOI:** 10.47626/1679-4435-2022-869

**Published:** 2023-08-08

**Authors:** Luciana Alves Mangueira, Mirella de Oliveira Guedes, Claudia Regina Guntzel, Marina Souza Vasconcelos, Tiago Veloso Neves

**Affiliations:** 1 Curso de Bacharelado em Medicina, Instituto Tocantinense Presidente Antônio Carlos, Palmas, TO, Brazil; 2 Centro de Referência em Saúde do Trabalhador, Secretaria Municipal de Saúde de Palmas, Palmas, TO, Brazil

**Keywords:** occupational exposure, occupational accidents, occupational health, exposição ocupacional, acidentes de trabalho, saúde do trabalhador

## Abstract

**Introduction:**

An occupational accident involving exposure to biological material is characterized by worker’s contact with organic fluids during the working day and in Brazil it is considered a condition of compulsory declaration.

**Objectives:**

To analyze the profile of victims of occupational accident involving exposure to biological material in the city of Palmas, state of Tocantins, from 2010 to 2020.

**Methods:**

Descriptive cross-sectional study using the Notifiable Diseases Information System.

**Results:**

During the study period, there were 1,173 notifications, most of which were female (80.10%), aged from 20 to 49 years (90.20%), and worked as statutory civil servants (44.50%). Percutaneous exposure was the most common type of accident, and blood was the main route of contamination (81.59%). Most victims evolved with discharge without serological conversion (66.92%), and 18.5% withdrew follow-up. The most frequent circumstance was improper disposal on the floor (12.45%). Health care professionals were the most involved in occupational accidents with biological material (79.02%) compared with professionals from other areas, with a predominance of nursing professionals (60.35%).

**Conclusions:**

The findings of this study reiterate training in the prevention of occupational accidents involving exposure to biological material is necessary for all jobs involving exposure to this type of material, regardless of the area, as well as changes in work organization so as to reduce the presence of risk factors for this type of injury.

## Abstract (pt)

**Introdução:**

O acidente de trabalho com exposição a material biológico caracteriza-se pelo contato do trabalhador com fluidos orgânicos durante a jornada laboral, sendo no Brasil considerado um agravo de notificação compulsória.

**Objetivos:**

Analisar o perfil das vítimas de acidente de trabalho com exposição a material biológico no município de Palmas, estado do Tocantins, entre os anos de 2010 e 2020.

**Métodos:**

Estudo descritivo de caráter transversal realizado utilizando dados do Sistema de Informação de Agravos de Notificação.

**Resultados:**

Nesse período houve 1.173 notificações, sendo a maioria das vítimas do sexo feminino (80,10%), com idade entre 20 e 49 anos (90,20%), e grande parte servidores públicos estatutários (44,50%). O acidente com exposição percutânea foi o tipo mais comum, sendo o sangue a principal via de contaminação (81,59%). A maioria das vítimas evoluíram com alta sem conversão sorológica (66,92%), e 18,5% abandonaram o acompanhamento da evolução. A circunstância mais frequente foi o descarte inadequado no chão (12,45%). Os profissionais de saúde foram os mais acometidos por acidentes de trabalho com material biológico (79,02%) em comparação com profissionais de outras áreas, com predomínio da categoria da enfermagem (60,35%).

**Conclusões:**

Os achados do presente estudo reiteram que a capacitação para a prevenção de acidentes de trabalho com exposição a material biológico se faz necessária a todas as funções com exposição a tal material, independentemente da área, bem como mudanças na organização do trabalho no intuito de reduzir a presença de fatores de risco para esse tipo de agravo.

## INTRODUCTION

An occupational accident (OA) involving exposure to biological material (BM) occurs when workers have contact with blood and/or other body fluids during their working day. The most frequent exposures result from percutaneous inoculation and direct contact with skin and/or mucosas.^1^ The risk of accidents varies according to the type of exposure, severity, size of injury, presence and volume of blood involved, clinical conditions of the source, and the prophylactic measures applied.^2^

The nursing staff is especially more susceptible to the occurrence of these exposures, due to the great number of workers in this professional category, to the constant handling of sharp objects, and to the constant provision of direct care to patients with several diseases.^3^

After an OA with BM, individuals should receive assistance appropriate to the type of occurrence, and the institution is responsible for referring them to care. Furthermore, workers need to receive prophylactic measures as briefly as possible, in order to minimize the risk for transmission of diseases such as HIV and hepatitis B.^4^

Hepatitis A vaccine is available for free through the public health network and confers immunity from 90 to 95% in immunocompetent adults, being the main pre-exposure prevention measure. However, nearly 25% of professionals are not vaccinated and are at increased risk of acquiring the disease in case of direct contact with contaminated BM.^5^

Therefore, isolated actions to prevent accidents are considered ineffective; thus, a combination of several strategies is necessary, namely: improvement of working conditions, especially work organization, in order to prevent fatigue; provision of personal protective equipment (PPE) and material of safety devices; and training sessions to promote changes in professionals’ behavior.^6^

In Brazil, Ordinance no. 1,271, of June 6^th^, 2014, which established the national list of diseases, injuries, and public health events of compulsory notification in public and private health services throughout the entire national territory, defined OA involving exposure to BM as a condition of compulsory notification.^7^

Knowledge of the profile of victims of OA involving exposure to BM allows for previous identification of main risk factors and enables to improve preventive actions, targeting mainly at the most susceptible groups. Therefore, this study aimed to analyze the profile of victims of OAs involving exposure to BM in the city of Palmas, state of Tocantins, Brazil, so as to quantify data available on the forms of the Notifiable Diseases Information System (Sistema de Informação de Agravos de Notificação, SINAN), delineate the profile of victims of these accidents, and evaluate the possible risk factors for OAs with BM.

## METHODS

This is a descriptive, cross-sectional study^8^ that used notifications forms available on the SINAN reporting OAs involving exposure to BM that occurred from 2010 to 2020, collected in June 2021. The reference population was all workers with notified cases living in the city of Palmas, state of Tocantins, Brazil. The characterization of victims of OAs involving exposure to BM included variables such as: age, sex, schooling, municipality of residence, occupation, working situation, type of exposure, organic material, circumstance of the accident, agent, use of PPE, and number of deaths.

This study was approved by the Research Ethics Committee of Fundação Escola de Saúde Pública de Palmas, through opinion number 4,677,466.

Inclusion criteria were all professionals who suffered an OA involving exposure to BM in the city of Palmas, Tocantins, reported to the SINAN from 2010 and 2020. Exclusion criteria were notification records with insufficient information for the study.

## RESULTS

Over the time interval analyzed, 1,173 cases of OAs involving exposure to BM were notified to the SINAN, of which 940 (80.10%) occurred in women. The most affected age group was from 20 to 49 years, accounting for 1,058 (90.20%) cases. With regard to the year of notification, there were no relevant variations, with the lowest number of cases being observed in 2013 (85; 7.20%), and the highest in 2017 (132; 11.30%) (Table l).

**Table 1:** Socio-demographic characteristics of professionals who suffered occupational accidents involving exposure to biological material in Palmas, state of Tocantins, Brazil, notified to the Notifiable Diseases Information System (Sistema de Informação de Agravos de Notificação, SINAN) from 2010 to 2020

Variable/category	Absolute frequency	Relative frequency (%)
Sex		
Male	233	19.9
Female	940	80.1
Total	1,173	100.0
Age group (years)		
< 1	0	0.00
1-4	0	0.00
5-9	0	0.00
10-14	1	0.10
15-19	16	1.40
20-34	590	50.30
35-49	468	39.90
50-64	96	8.20
65-79	2	0.20
> 80	0	0.00
Total	1,173	100.00
Year of notification		
2010	101	8.60
2011	112	9.50
2012	97	8.30
2013	85	7.20
2014	115	9.80
2015	89	7.60
2016	108	9.20
2017	132	11.30
2018	111	9.50
2019	98	8.40
2020	125	10.70
Total	1,173	100.00

Source: SINAN

Occupational data showed that statutory civil servants and registered employees accounted for the highest number of cases, with 522 (44.50%) and 303 (25.83%) notifications, respectively (Table 2).

**Table 2:** Occupational data of professionals who suffered an occupational accident involving exposure to biological material in Palmas, state of Tocantins, Brazil, notified to the Notifiable Diseases Information System (Sistema de Informação de Agravos de Notificação, SINAN) from 2010 to 2020

Variable/category	Absolute frequency	Relative frequency (%)
Working situation		
Unknown/blank	23	1.96
Employee with formal contract	303	25.83
Employee without formal contract	23	1.96
Self-employed	29	2.47
Statutory civil servant	522	44.50
Civil servant with formal contract	160	13.64
Retired	0	-
Unemployed	2	0.17
Temporary worker	16	1.36
Cooperative member	2	0.17
Freelance worker	0	-
Employer	0	-
Other	93	7.93

Source: SINAN.

In relation to the accidents, blood was found to be the main route of contamination with organic material, being present in 947 (81.59%) of accidents. Furthermore, 785 (66.92%) victims evolved to discharge without serological conversion, 116 (9.89%) were discharged with negative serology, 217 (18.50%) patients withdrew follow-up, and 23 (1.96%) had incomplete or blank data, with no reports of either deaths or serological conversion. The most frequent circumstance was improper disposal on the floor (146; 12.45%), with values similar to those of improper garbage disposal (129; 11%), other (120; 10.23%), D-glucose test (119; 10.14%), and administration of intravenous medication (111; 9.46%) (Table 3).

**Table 3:** Characteristics of occupational accidents involving exposure to biological material in Palmas, state of Tocantins, Brazil, notified to the Notifiable Diseases Information System (Sistema de Informação de Agravos de Notificação, SINAN) from 2010 to 2020

Variable/category	Absolute frequency	Relative frequency (%)
Case evolution		
Unknown/blank	55	4.69
Discharge with serological conversion	0	-
Discharge without serological conversion	785	66.92
Discharge of source patient with negative serology	116	9.89
Withdrawal	217	18.50
Death caused by the accident	0	-
Death from another cause	0	-
Circumstance of the accident		
Unknown/blank	3	0.26
Administration of intravenous medication	111	9.46
Administration of intramuscular medication	67	5.71
Administration of subcutaneous medication	44	3.75
Administration of intradermal medication	5	0.43
Puncture collection	59	5.03
Non-specified Puncture	60	5.12
Improper waste disposal	129	11.00
Improper disposal on the floor	146	12.45
Laundry	6	0.51
Material washing	46	3.92
Handling box with sharp material	61	5.20
Surgical procedure	84	7.16
Dental procedure	39	3.32
Laboratory procedure	52	4.43
D-glucose test	119	10.14
Recapping	22	1.88
Other	120	10.23
Organic material		
Unknown/blank	7	0.60
Blood	957	81.59
Cerebrospinal fluid	10	0.85
Pleural fluid	1	0.09
Ascitic fluid	1	0.09
Amniotic fluid	1	0.09
Fluid with blood	100	8.53
Serum/plasma	11	0.94
Others	85	7.25

Source: SINAN.

Health care professionals were the most involved in OAs with exposure to BM, accounting for 79.02% of the cases, with a predominance of nursing professionals (nursing attendant, nursing assistant, nurse, surgical nurse, nursing technician, and family health strategy nursing technician), who accounted for 60.35% of the total number of cases, followed by dentists and other dental professionals (dental office assistant, denturist, oral health assistant in the family health strategy, and dental hygiene technician) (62; 5.31%) and physicians (59; 5.06%).

The sample also included interns (34; 2.90%), physical therapists (22; 1.88%), personnel assistants (25; 2.13%), domestic servants in general services (24; 2.05%), laboratory assistants (18; 1.53%), construction industry professionals (workers in building and public spaces maintenance areas, and bricklayer) (6; 0.52%), beauty/esthetics professionals (hairdresser, beautician, and manicure) (10; 0.86%), and education professionals (pharmacy and biochemistry teacher, educator) (2; 0.18%).

Additionally, OAs were also reported in cleaning/maintenance professionals (recyclable material collector, chambermaid, garbage collector, laundry assistant, collector of solid waste from health services, hospital kitchen assistant, janitor, street cleaner, gardener, washer person, clothes washer person, dry cleaner, fish preparation worker, building janitor, domestic servant, and plumber) (90; 7.70%), pharmacy professionals (drugstore clerk and pharmacist) (15; 1.28%), professionals of other different areas (undertaker, artist, police officer specialized in dactyloscopy, and student) (37; 3.17%), and public/private security professionals (security agent, prison security agent, army officer, and military police soldier) (13; 1.12%).

Finally, administrative/executive/office professionals (information analyst, administrative assistant, office assistant, receptionist, executive secretary, administrative supervisor, staff assistant, hotel manager, responsible for mechanical maintenance of operating systems) (41; 3.52%) and laboratory professionals (clinical analysis laboratory assistant, pharmacy laboratory technical assistant, biomedical doctor, physico-chemical analysis laboratory technician, industrial laboratory technician, pharmacy laboratory technician, biotechnologist, clinical pathology technical assistant, biochemical pharmacist, and clinical pathology technician) (49; 4.20%) were also involved in OAs (Table 4).

**Table 4:** Victims of occupational accidents involving exposure to biological material in Palmas, state of Tocantins, Brazil, notified to the Notifiable Diseases Information System (Sistema de Informação de Agravos de Notificação, SINAN) from 2010 to 2020, according to occupation/professional category

Category	Absolute frequency	Relative frequency (%)
Community health worker	4	0.34
Biologist	2	0.17
Dental professional	62	5.31
Waste collector	33	2.82
Hospital kitchen assistant	1	0.09
Nursing professional	708	60.36
Physical therapist	22	1.88
Speech therapist	1	0.09
Scrub nurse	7	0.60
Physician	59	5.06
Driver	8	0.69
Construction industry professional	6	0.52
Beauty/esthetics professional	10	0.86
Educational professional	2	0.18
Cleaning/maintenance professional	90	7.70
Pharmacy professional	15	1.28
Professionals of other different areas	37	3.17
Public/private security professionals	13	1.12
Administrati ve/executi ve/off ice professionals	41	3.52
Laboratory professional	49	4.20
Clinical psychologist	1	0.09
Orthopedics technician	1	0.09
Radiology and imaging technician	1	0.09
Total	1173	100.00

Source: SINAN.

## DISCUSSION

The present study observed that most OAs involving exposure to BM occurred in women (80.10%), a fact that results from the great participation of women in the health workforce, more specifically in the nursing area, which accounts for the highest number of professionals in hospitals and is one of the professional categories that has direct contact with patients on a more frequent basis. These findings are similar to those of a study conducted in the macroregion of Florianópolis, state of Santa Catarina, Brazil, which also found a predominance of females in notifications of OAs involving exposure to BM.^9^ Moreover, there was a predominance of individuals aged from 20 to 59 years (90.2%), covering the period when people enter the labor market up to their retirement.

Blood was the most commonly identified material in accidents (81.59%), since it is handled for a variety of tests. The risk for contamination with diseases such as hepatitis B, hepatitis C, and HIV is considered low,^10^ a fact that can be confirmed by the percentage of victims who evolved to discharge without serological conversion (66.92%). However, a considerable number (18.50%) of patients withdrew follow-up and, since these highly severe diseases can result in professional’s death or impair their capacity, it is necessary to show the importance of monitoring the case until its resolution, making proper use of PPE, and providing courses and accessible information on disposal and handling of materials.

Accidents with sharp materials were the most reported type of OA, and improper disposal, D-glucose test, and administration of intravenous medication accounted for 46.91% of circumstances of accidents. This percentage suggests a possible lack of professionals’ training and health responsibility, since previous studies showed that many professionals are not aware of health regulations for disposal and capacity of containers.

Furthermore, routine work in environments with poor structure may contribute to reduce professionals’ perception of risk.^11^ Use of inadequate collectors and inadequate use of PPE as factors influencing these numbers, as well as organizational factors such as teams with a limited number of workers, demand disproportionate to the size of the team, stressful work environment, lack of appropriate rest at the workplace, since all these factors make workers more vulnerable to an OA involving exposure to BM. It is known that professionals who handle BM are more often in the nursing area, thus justifying the fact that 79.02% of accidents involved nursing professionals.^12,13^

With regard to occupation, health care professionals account for the vast majority of the cases. This result was expected, since the main type of exposure was percutaneous, the most common route was blood, and the most frequent circumstance was improper disposal of sharps. Considering that other professionals do not have, or have minimum contact with these factors, the number of accidents involving exposure to BM becomes small compared with that observed among nursing professionals, for example, who handle sharps on a routine and common basis.^14^

Furthermore, individuals who are not health care professionals, especially garbage collector, work in unhealthy environments. In most cases, there is no distribution of PPE, work has few rest intervals, there is constant noise, work rhythm is fast, hygiene is poor, and many workers are not concerned about proper discard of materials that may cut or cause perforations.^15^

Considering the data shown in this study, measures should be adopted to prevent accidents. In Brazil, Ordinance no. 485, of November 11^th^, 2005, which defines the standards for regulating occupational safety and health, sets for the distribution of PPE in sufficient number and its proper use by professionals.^16^ It is also crucial to value professional training, focused on knowledge of techniques for proper and safe handling of sharps, in addition to proper disposal; other measures include promotion of hepatitis B vaccination to all health care professionals, since it available for free and often confers sufficient immunity; inspection of compliance with the agreed working hours and with rest intervals, thus preventing workers’ burnout; and adequacy of work environments, providing proper infrastructure and hygiene.

## CONCLUSIONS

Most workers involved in OA with exposure to BM were health care professionals working as statutory civil servants. This position provides them with job stability, but with fewer wage changes, which may lead servants to work in more than one job, to take long work hours, and to work in night shifts, thus influencing in their risk of accidents. It is necessary to reduce the cases of OA involving exposure to BM, to reduce the number of invasive procedures (as much as possible), and to promote a safe work environment and an adequate ratio between health personnel and the population of patients assisted.

Prevention measures should include joint actions, established between workers and service managers, so as to improve working conditions, especially work organization, to supply materials with safety devices, to repeat serological tests for HIV and hepatitis B and C viruses, and to raise awareness with the purpose of promoting behavioral changes among workers and managers, because isolated actions are considered ineffective to minimize these problems. Moreover, it is necessary to improve the structure of the services responsible for care provision to injured individuals, and also provide full support from exposure to discharge.

Outcomes were favorable, with no notifications of deaths or serological conversion. However, it is worth highlighting the significant percentage of follow-up withdrawal, evidencing the need of searching for new strategies among the involved sectors in order to reduce these percentages, with the understanding that withdrawal is a multifactorial event. It is also worth observing the need of qualifying the staff responsible for registering information, because it has an impact on investigation, monitoring, and follow-up of worker’s health.
